# Blood‐Brain Barrier Dynamics in Vascular Dementia: Unravelling the Tripartite Crosstalk between the Endothelium, Pericytes, and Microglia

**DOI:** 10.1002/alz.087126

**Published:** 2025-01-03

**Authors:** Axel Montagne

**Affiliations:** ^1^ UK Dementia Research Institute, University of Edinburgh, Edinburgh United Kingdom

## Abstract

**Background:**

Small vessel disease (SVD) is a disorder of the brain’s microvessels and a common cause of dementia and stroke. Evidence links normal ageing features to SVD progression, involving endothelial activation, pericyte dysfunction, BBB failure, and microglia response. Here, we aim to examine this relationship through a series of translational investigations.

**Method:**

Using microscopy, we quantified pericyte coverage and endothelial VCAM‐1 expression in young/aged mice and control/SVD cases. Pericyte and endothelial fluid biomarkers were analysed in a mild stroke cohort, correlating with SVD MRI markers. We employed a novel AAV targeting brain endothelial cells to overexpress (OE) *Vcam1*, *Icam1*, *Selp* and *Sele* in mice, shedding light on endothelial activation’s role in pericyte and BBB dysfunctions, but also the reciprocal influences with the microglial response. Finally, we genetically manipulated brain pericytes using our novel *Atp13a5‐CreER* mouse model, offering insights into their biological impact on endothelial and BBB functions.

**Result:**

Aged mice and SVD cases showed reduced pericyte coverage and increased VCAM‐1 expression in microvessels (<10 µm). Biomarker analyses revealed associations between PDGFRB, PDGF‐BB, ICAM‐1, VCAM‐1, and E‐selectin concentrations with specific SVD manifestations. AAV experiments highlighted correlations between endothelial activation, pericyte dysfunction, reduced brain perfusion, and BBB breakdown, with distinct immune responses observed in AAV‐*Vcam1^OE^* and AAV‐*Icam1^OE^* groups. The *Atp13a5‐CreER* model unveiled diverse pericyte subtypes and their pro‐inflammatory impact on capillary endothelial cells.

**Conclusion:**

Our approach underscores endothelial‐pericyte crosstalk’s crucial role in dementia. Integrating post‐mortem tissues, fluid biomarkers, viral manipulations, and mouse models enriches understanding, guiding potential therapeutic interventions in vascular dementia.

